# Supplementary material for: Hyperbilirubinemia in Gunn Rats Is Associated with Decreased Inflammatory Response in LPS-Mediated Systemic Inflammation
Source: Int J Mol Sci. 2019 May 9;20(9):2306. doi: 10.3390/ijms20092306 (PMC6539717; doi:10.3390/ijms20092306)
Supplement: Supplementary file 1 [file ijms-20-02306-s001.pdf]

Supplementary Figures

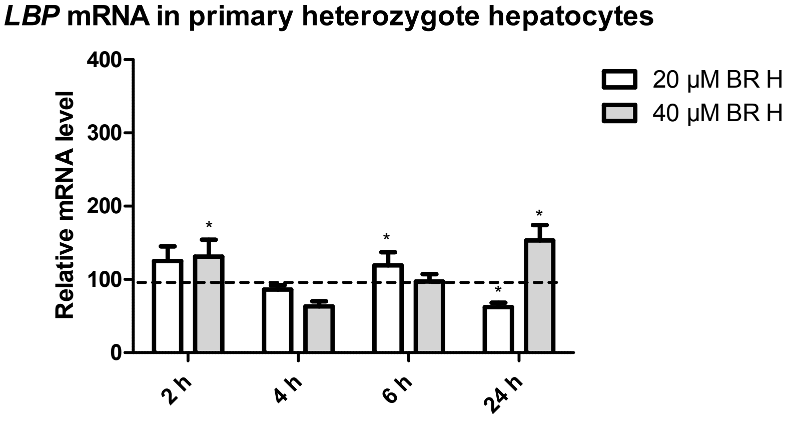

Figure S1. The effect of BR on LBP mRNA expression in the primary hepatocytes. Primary hepatocytes isolated from normobilirubinemic heterozygous controls were incubated with BR (20 and 40 µM) for 2, 4, 6 and 24 h. Values are expressed % of untreated control cells (100%).*p<0.05 vs. controls. n=6 independent cell cultures per group.

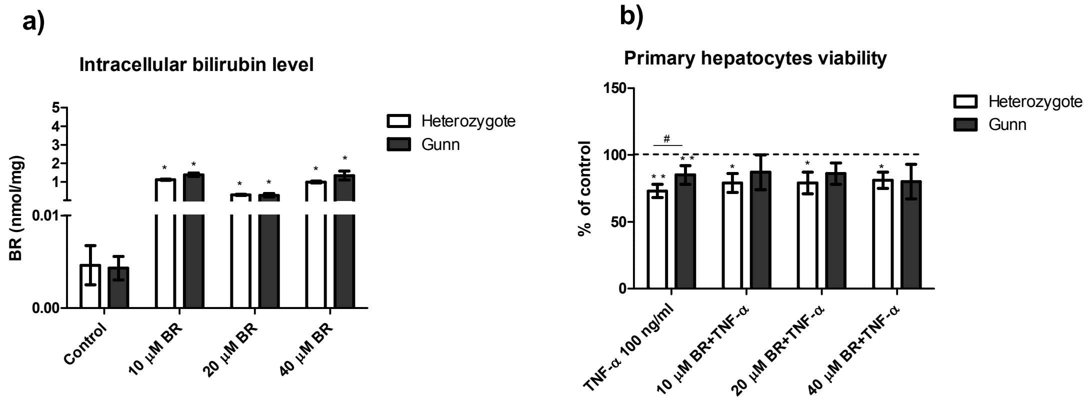

Figure S2. The effect of BR and TNF-α on viability of primary hepatocytes. Intracellular BR levels (a) and cell viability (b) were measured 24 h before and after BR and TNF-α treatment in primary hepatocytes isolated from normobilirubinemic heterozygous controls (Heterozygote) and hyperbilirubinemic Gunn rats (Gunn), respectively. *p<0.05 vs. corresponding control, #p<0.05 vs. heterozygote primary hepatocytes. (a) n=6, (b) n=10 independent cell cultures per group.

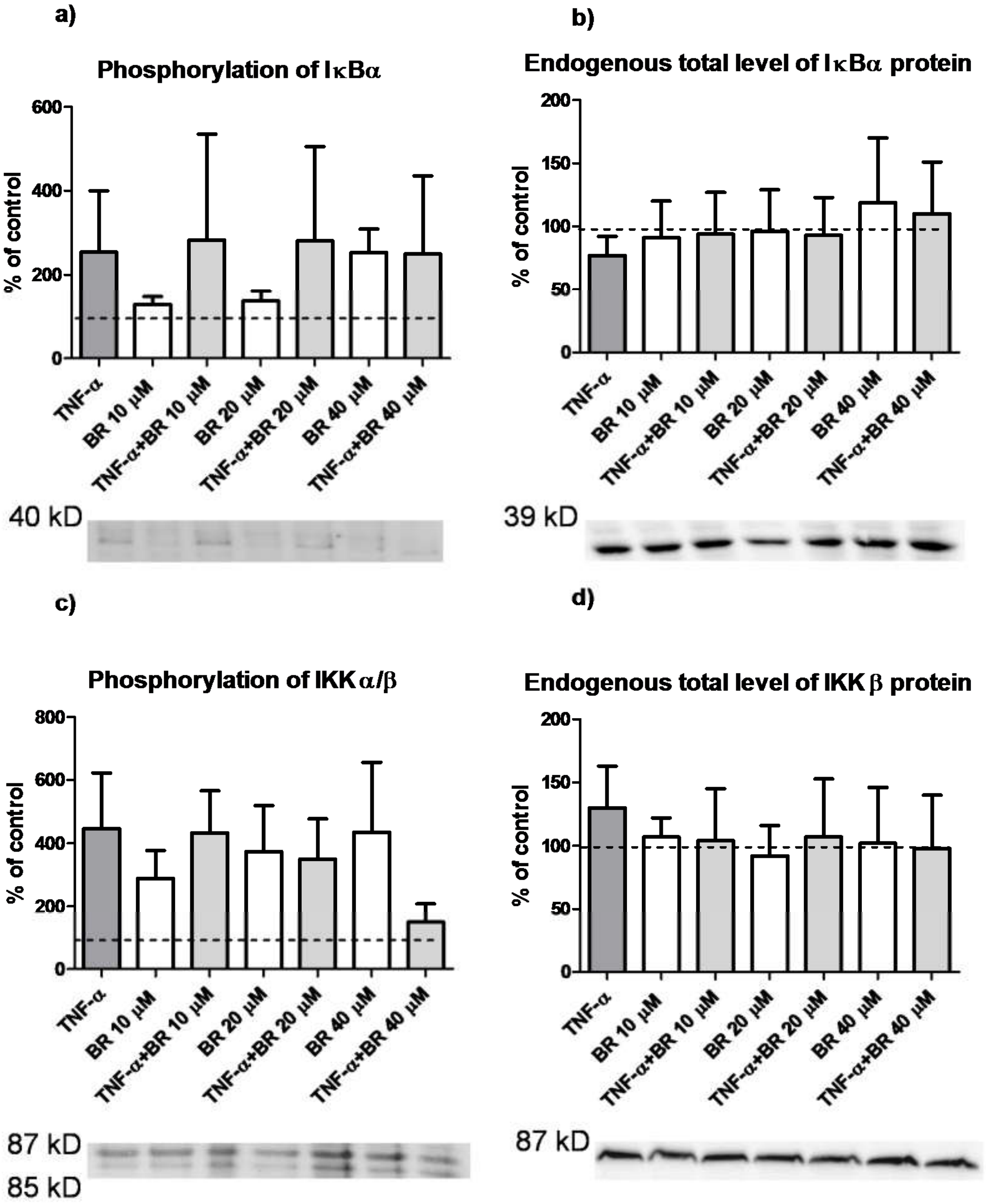

Figure S3. The effect of bilirubin on NF-κB signaling pathway. Both types of primary hepatocytes were pre-incubated with BR (0-40 μM) for 2 h and then treated with TNF-*α* (12 ng/ml) for 5 min. Total (b) IκB*α*, (d) IKKβ and phosphorylated (a) IκB*α* and (c) IKK*α*/β were measured by Western blot. Values are expressed as % of untreated control cells (100%). *$p<0.05$ vs. TNF-*α*. n=6 independent cell cultures per group.
